# In-Line Sorting of Harumanis Mango Based on External Quality Using Visible Imaging

**DOI:** 10.3390/s16111753

**Published:** 2016-10-27

**Authors:** Mohd Firdaus Ibrahim, Fathinul Syahir Ahmad Sa’ad, Ammar Zakaria, Ali Yeon Md Shakaff

**Affiliations:** 1 Center of Excellence for Advanced Sensor Technology (CEASTech), Universiti Malaysia Perlis (UniMAP), Taman Muhibbah, Jejawi, Arau, Perlis 02600, Malaysia; fathinul@unimap.edu.my (F.S.A.S.); ammarzakaria@unimap.edu.my (A.Z.); aliyeon@unimap.edu.my (A.Y.M.S.); 2 School of Mechatronics Engineering, Universiti Malaysia Perlis (UniMAP), Pauh Putra Campus, Arau, Perlis 02600, Malaysia; 3 School of Bioprocess Engineering, Universiti Malaysia Perlis (UniMAP), Kompleks Pusat Pengajian Jejawi 3, Kawasan Perindustrian Jejawi, Arau, Perlis 02600, Malaysia

**Keywords:** machine vision, mango, Fourier descriptor, disk method, shape, volume estimation, image processing, mass estimation

## Abstract

The conventional method of grading Harumanis mango is time-consuming, costly and affected by human bias. In this research, an in-line system was developed to classify Harumanis mango using computer vision. The system was able to identify the irregularity of mango shape and its estimated mass. A group of images of mangoes of different size and shape was used as database set. Some important features such as length, height, centroid and parameter were extracted from each image. Fourier descriptor and size-shape parameters were used to describe the mango shape while the disk method was used to estimate the mass of the mango. Four features have been selected by stepwise discriminant analysis which was effective in sorting regular and misshapen mango. The volume from water displacement method was compared with the volume estimated by image processing using paired *t*-test and Bland-Altman method. The result between both measurements was not significantly different (P > 0.05). The average correct classification for shape classification was 98% for a training set composed of 180 mangoes. The data was validated with another testing set consist of 140 mangoes which have the success rate of 92%. The same set was used for evaluating the performance of mass estimation. The average success rate of the classification for grading based on its mass was 94%. The results indicate that the in-line sorting system using machine vision has a great potential in automatic fruit sorting according to its shape and mass.

## 1. Introduction

External quality of an agricultural product is the first criterion used by consumers beforehand in determining its eating quality. External quality can be described as the quality of the fruit as described by its color, texture, size, shape and visual defects [1]. These parameters are the proper standards for grading and classifying of the product before sale to the consumer. Harumanis mango—which is the most famous fruit at Perlis Province in Malaysia—has a very high value in the export market. The increasing demand from local and overseas consumers requires the process of classifying and sorting the mango to be more stable, accurate, non-destructive and fast. The current method of weighing Harumanis mango is done manually using a scale, while classifying the bad and normal shape was done by human labor. This method proves to be time-consuming, inefficient and labor intensive. 

There are two common methods of measuring the volumes of agricultural products and foodstuffs [2]. First is the liquid displacement method—which is the easiest and simplest way. The object will be immersed in liquid and the displacement will be measured, but the product may be damaged during the process. This method is inaccurate, especially for fragile or porous objects [3,4]. The gas displacement method preserves the condition of the food and leaves it intact, but it is not suitable for a fast measurement system. Most fruits have an irregular shape, but they can be described as having a standard shape such as round, oblong, and elliptical. The uniformity of the presumed shape of the fruit will determine the accuracy of the estimated volume. Model equations were developed to estimate the volume of citrus and kiwi fruit by using the length and major and minor diameters [5,6]. Another model was developed using geometrical features to estimate the mass of mangoes (cv. ‘Manila’) [7]. The predicted value and the measured value have a high correlation (*R*^2^ = 0.93). However, both methods used mechanical means to measure the required dimensions. The measurement methods were subject to human error and an inefficient approach for sorting a large quantity of product.

A classifying system which can rapidly calculate the volume is needed by the commercial sorter industry. The system also needs to be cheap and easy to implement in the available machinery. Structured light projection [8,9] has the potential to give accurate estimated volumes. However, it needs extra hardware to be interfaced with the computer, leading to additional high costs. Machine vision has gained much interest from many researchers in determining the volume and mass of agricultural products. Forbes developed a machine vision algorithm using a neural network to estimate the volume of pears [10]. The RMS percentage error was slightly reduced when multiple images were used compared to the single image analysis. Another method to obtain the volume is by using the ellipsoidal method. Omid [11] divided citrus fruit images into numbers of elliptical frustums. The volume then calculated by summing all the individual volume frustums. The results show that the computed volume has some good agreement with the actual volume. Koc [12] also used this method to determine the volume of watermelons. Both methods used samples that can be categorized as spherical shapes, thus only one dimension was measured and use to estimate the volume. Chalidabhongse [13] used multiple cameras to capture multiple silhouette images of mango. These images were then used to obtain a 3D volume reconstruction of the mango. However, the experiment setup can only be applied as an offline system.

Shape is another factor that mostly been used to measure the quality of fruit during the grading and classification process. This process is usually done in the packing house, where the graders will judge the fruit shape base on the difference from the normal characteristic shape of the variety. Abnormality in shape mostly leads to rejection of foreign consignments [14]. Misshapen and malformation are the most common words to describe a product’s poor symmetry and abnormal concavities [15]. A fast and effective machine vision system is needed to grade the Harumanis mango. There are two main categories to characterize fruit shape which are size dependent measurements and size independent measurements [14]. Size-dependent measurements are a combination of size measurement features such as roundness, elongation, eccentricity and others. The combination will form a descriptor which can describe the regularity of the fruit shape. For size independent measurements, boundary-based and region-based method is used to analyze the shape of the fruit. Some methods developed using this concept include the Fourier descriptor (FD), Chain code and Wavelet descriptor. The Fourier descriptor method mostly been used to obtain the shape characteristics of fruit because it is computationally inexpensive, robust and each of its FD have meaning [16].

The objective of this research was to develop a rapid, real-time automatic classification and grading method for Harumanis mango using machine vision based on its shape pattern and estimated mass. A conveyor machine was used to transport the mango to each station. An algorithm was developed to grab the image and analyzed the shape, volume, and mass of the mangoes on the in-line system. Mango with defective shapes will be classified as reject fruit while the normal shaped fruit will be graded into three categories according to its volume. The system has been fully tested in the Perlis Department of Agriculture to grade their Harumanis mango production.

## 2. Materials and Methods

One hundred and eighty Harumanis mangoes were randomly picked at an orchard managed by the Perlis Department of Agriculture, Malaysia. All the mangoes picked had been harvested in the past 24 h. Each sample was measured using a caliper to obtain its dimensions including maximum length, maximum wide and maximum height. The measurement was taken with an error of ±1.0 mm. The measurements were used to compare the image measurements with the real measurements. Each mango was weighed using a precision digital scale (PL1502E portable balance, Mettler Toledo, Greifensee, Switzerland) with ±5 g accuracy. The water displacement method was used to measure the actual volume of each mango. A square container was filled full with water. The mangoes were submerged into the container and the volume of water displaced by the mango was measured using a 1000 mL beaker. 

### 2.1. Element of Machine Vision

**Figure 1:**
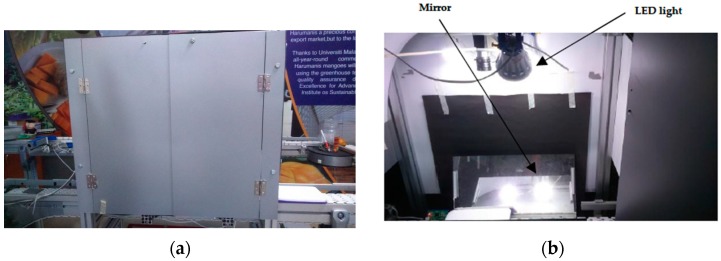
(**a**) Main frame of the image capturing platform; (**b**) Camera, light bulb and mirror setup inside the platform.

The image capturing platform as shown in Figure 1a consists of a rectangular box made from PVC-coated aluminum with dimension of 100 cm × 60 cm × 100 cm, a CMOS camera (a 2 megapixel acA1600-60gc digital camera with 4.5 µm × 4.5 µm pixel size, Basler, Ahrensburg, Germany) with a GigE interface and four LED light sources (20 W). A lens with 8 mm focal length was attached to the camera. A computer of 8 GB RAM memory and 3.2 GHz processing speed was installed with the LabVIEW 2012 (National Instruments, Austin, TX, USA) visual programming language. The platform was placed on a conveyor system. The box was made to ensure fully isolated conditions which prevent outside lighting. The camera was mounted perpendicular to the conveyor surface with a height of 45 cm above the conveyor. With the GigE interface, images’ raw data were packed and send through the Ethernet cable to the computer with speeds up to 1000 Mb/s. After a data packet had been received, the NI-IMAQdx driver extracted the image and passed it to the program. The entire light source mounted on top of the box with the direction facing downward to the conveyor surface. Figure 1b shows the setup inside the platform. The direction was adjusted to avoid any bright spots and specular reflection on the mango. LED bulbs were used rather than compact fluorescent (CFL) bulbs because they did not produce any flicker effect. This allowed each captured image to have a constant luminance which simplifies the process of identifying and analyzing the mango. A plane reflected mirror was mounted on the conveyor with the angle of 65° from the conveyor surface. This mirror provides the side view of the mango, hence allowing the side view to be captured by the camera.

Each mango was placed on white background tray which enhanced the contrast between the object and the background. These trays traveled from loading area to capturing platform and finally to the sorting area by means of a conveyor. The conveyor was designed with a closed loop rectangular path system. The camera has a frame rate up to 60 frames per second (fps) which allows the image to be captured as if it was in a static state. Movement of the conveyor and the actuator was controlled by the computer using the Compact-RIO module (National Instruments, Austin, TX, USA). 

**Figure 2:**
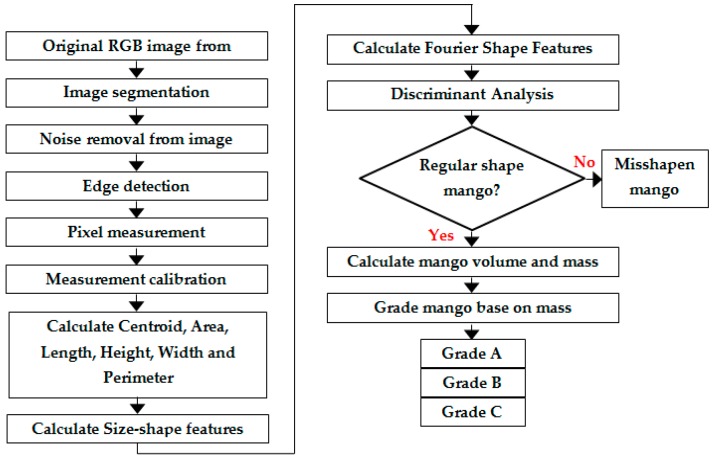
The methodology involved in this research.

A data flow programming language or known as G-language was developed along with the graphical user interface (GUI) to control the movement of the conveyor and capture the images of the mangoes. The program was constructed and written specifically for this application by optimizing the parallel programming capability of the software to control the hardware and process the data simultaneously. All important steps involved in detecting shape regularity and volume estimation of mango are shown in Figure 2. The first step was for image acquisition and pre-processing. The second step was for pixel measurement which then used for measurement calibration. The third step was for calculating the physical features extraction such as centroid, area, length, width, height and perimeter. The fourth step was for calculating the size-shape features and Fourier-shape features. Two most important steps were the stepwise discriminant analysis stage and the volume and mass estimation stage. Discriminant analysis was used to select the most important features that can determine the shape regularity of the mango. The misshapen shape mangoes were classified as reject while the regular shape mangoes were analyzed again to determine its volume and mass. Then the mangoes were graded into three grades according to their mass. 

### 2.2. Image Acquisition

Each tray has a RFID card which was used for tray identification and data validation at every station. As soon as a tray is detected by the RFID reader at the image capturing platform, the program will trigger the camera to capture the images. The 2-megapixel camera can capture a high-quality image with a resolution of 1600 pixels × 1200 pixels. The drawback of the high-quality image was the time taken for the data to be transferred to the computer—which can take up to 1 s. This duration is intolerable when developing a rapid online system. By reducing the resolution to 1600 pixels × 850 pixels, it only takes 300 ms to capture and transfer the image data packets. 

**Figure 3:**
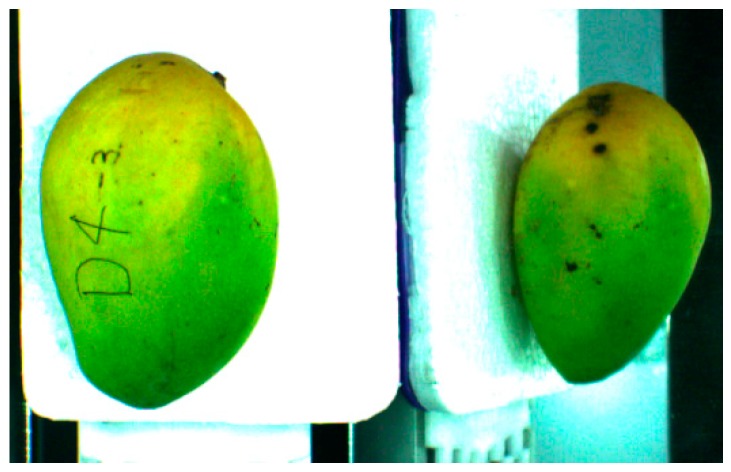
The top view and side view of mango captured by the camera in a single image.

The conveyor did not stop during the acquisition, and the tray traveled along the conveyor with a speed of 0.8 m/s. In each acquisition, two objects were seen in the image which was the view from the top and the view from the side of the mango as in Figure 3. This was the major achievement of the system which reduced the usage of camera and time needed to obtain two different views at the same time. The side view of mango was obtained from the image reflected by the mirror. The information of each mango was stored in the buffer and tagged with the ID number of the RFID card in their respective tray.

### 2.3. Image Pre-Processing

**Figure 4:**
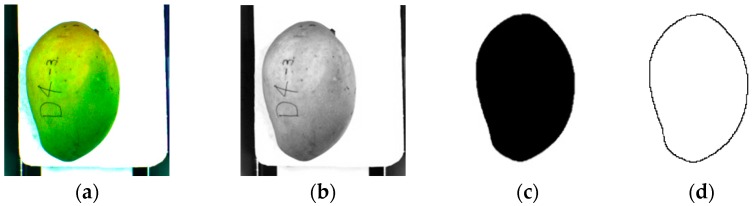
Image processing step involve in background removal for top view image. (**a**) Original RGB image; (**b**) Grayscale image from the B band; (**c**) Binary image after global threshold process; (**d**) Shape boundary of the mango.

At first, a segmentation process was done to separate the mango from the background. The image was split into two parts. The first part shows the top view of the mango while the second part shows the side view of the mango. The images need to be separated as the pre-processing method is different for each of them. The top view image of a mango has high contrast and very low noise. The white background has high R, G and B values in RGB coordinate while the mango has a low value of B. The original image was converted to a grayscale image by extracting the B value of the image as in Figure 4b. The B value has a range of 0 to 255. From the grayscale image, the multiple global threshold technique was used to eliminate the background and the stem [17]. Pixel values less than 90 were converted to 0 (black) and pixel values higher than 90 were converted to 255 (white) [12]. Another threshold value was selected to eliminate the stem, which pixel values in the range of 0 to 20 were converted to 255 (white). Dark spots and dirt on the mango, and the conveyor belt outside the tray area can produce some random noise on the image. A small particle removal method was used to remove any small connected components that have lower pixel values than a predefined value. This method only retained the large object which was the mango. An image fill holes algorithm was used to fill any holes inside the mango area which might form during elimination of the dark spot and dirt. The resulting image was in binary format image as in Figure 4c, where the white areas represent the background and the dark areas represent the object which is the mango. Figure 4d shows the shape boundary of the mango after a thinning process [18]. The Fourier descriptor method was then used to analyze the shape of mango for any abnormalities by using the thinning image.

**Figure 5:**
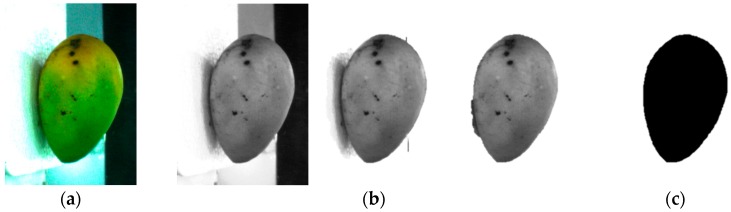
Image processing step involves in background removal for side view image. (**a**) Original RGB image; (**b**) Process of background removal by multi-stage global threshold; (**c**) Binary image.

The side view image has low contrast as it is the reflection from the mirror. Furthermore, some amount of shadow was seen from the side view as the mango obstructs the light from above. An additional pre-processing step was needed to eliminate the shadow and obtain the shape of the mango. Three repetitive global thresholds, first on the B band, second on G band and last on the B band with different threshold values were applied. A median filter of size 7 pixels × 7 pixels was used to remove the boundary of the shadow [19]. The binary image was used to crop the original images for the next global threshold step. Figure 5c shows the final image after the correction process.

### 2.4. Measurement Calibration

**Figure 6:**
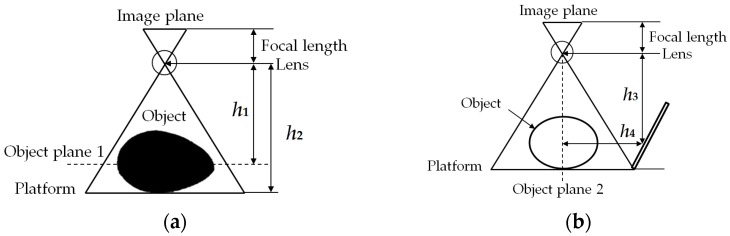
The concept of scale used for measurement calibration. (**a**) The model used for calibrating top view image; (**b**) The model used for calibrating side view image.

Measurement calibration was determined using the scale of aerial photograph model. Cohen [20] explained that the scale of aerial photograph is a function of the flying height of the camera and focal length of the camera. It is most referred to as the conversion between the number of units on the ground and the equivalent unit of distance on the photograph. This model can be applied in this research with some modification as the working method for both of them is similar. The model uses focal length of a lens to calculate the magnification of distant object with no image distortion. The equation model as in Equation (1), calculates the object size by using the product of image size (pixels), pixel size, and the distance between the camera lens and object plane then divides it by the focal length of the lens. Figure 6 shows the concept of scale magnification used in this research.

Object size = (Image size × Pixel size × Distance)/Focal length(1)

Koc [12] in his research indicated that as the size of the object increased, the volume estimated by the image processing was exaggerated. This phenomenon was caused by the change in distance of the object from the camera. Koc calibrated the measurement only once during his research which contributed to the error. In order to eliminate this error, the calibration was done in each image capturing process. The first step was to obtain the height of the mango from the side view image. The calibration for the side view image was done only once as the height between the camera and the mirror was constant as in Figure 6b. Mango placement was always in the center of the platform. One of the characteristics of the plane mirror is that the distance between the object and the mirror is identical to the distance between the image and the mirror. Hence, the reflected image was calibrated by combining the distance from the camera to mirror and mirror to object (*h*_3_ *+ h*_4_) to get the total distance. The result then was used to calculate the height of the object plane 1, which was the height of the major length of the mango. Distance from camera to the platform, *h*_2_ was deducted with the previous height to obtain the distance from camera to the object plane 1, *h*_1_. The final equation for each calibration is shown in Equations (2) and (3):

Object size _top_view_ = (Image size × Sensor size × *h*_1_)/Focal length (2)

Object size _side_view_ = (Image size × Sensor size × (*h*_3_*+ h*_4_))/Focal length (3)

Paired *t*-test and mean difference confidence interval approach were used to compare the length and height measured using image processing and caliper. The paired *t*-test was used to identify the significant difference between the caliper measurement and image processing method. Bland-Altman approach [21] was used to plot the agreement between both dimension measured by caliper and image processing. Both of the statistical analyses have also been used to find the difference and agreement of the volumes measured using water displacement and the disk method in the later stages of the research. The statistical analyses were performed using GraphPad Prism (GraphPad Software, Inc., La Jolla, CA, USA).

### 2.5. Shape and Fourier Features Extraction

There are many methods to analyze the shape of an object. Elmasry [22] stated that a combination of measurement features such as perimeter, area, width, and length are used in the conventional method of shape description. In this research, aspect ratio, area ratio, and roundness were used for features measurement. Equation (4) to Equation (6) show the formulas used to obtain the stated feature measurements:

Aspect Ratio = Max diameter/Min diameter(4)

Area Ratio = Area/(Max Diameter/Min Diameter)(5)

Roundness = (4π × Area)/(π × Max diameter^2^) (6)

Another method is using the Fourier descriptor (FD). This method has been used widely to analyze the shape of agricultural products. Agricultural products that have been using this method are including mango [23,24], potato [22] and pistachio nut [25]. The first step of FD is to find the two-dimensional centroid (X_c_,Y_c_) which is given by using mathematical equation as in Equations (7) and (8):

(7) $$x_{c}=\frac{\sum_{k=0}^{N}y_{k}\left(x_{k}^{2}-{\;x}_{k-1}^{2}\right)\;-{\;x}_{k}^{2}\left(y_{k}-{\;y}_{k-1}\right)}{2\sum_{k=0}^{N}y_{k}{(x}_{k}-x_{k-1})\;-x_{k}{(y}_{k}-{\;y}_{k-1})}$$

(8) $$y_{c}=\frac{\sum_{k=0}^{N}y_{k}^{2}\left(x_{k}-{\;x}_{k-1}\right){\;-\;x}_{k}\left(y_{k}^{2}{\;-\;y}_{k-1}^{2}\right)}{2\sum_{k=0}^{N}y_{k}{(x}_{k}{\;-\;x}_{k-1})-{\;x}_{k}{(y}_{k}{-\;y}_{k-1})}$$

**Figure 7:**
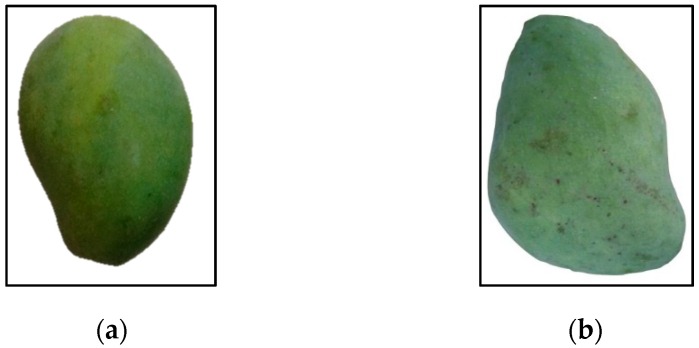
(**a**) Image of regular mango; (**b**) Image of misshapen mango with non-uniform edge.

**Figure 8:**
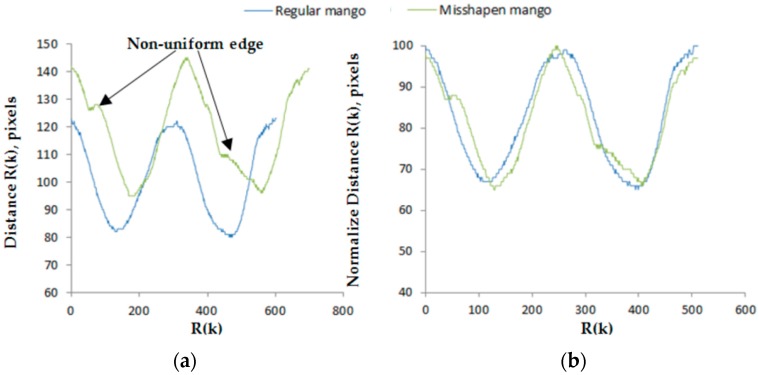
Distance signature of regular mango and misshapen mango with non-uniform edge. (**a**) Raw distance R(k) from the centroid to the boundary points; (**b**) Distance signature is normalized by 100 while number of distance point normalized to 512 points. It shows that the presence of non-uniform edge has affected the boundary profile.

*N* represents the total number of pixel boundaries which defined in a clockwise direction. (X_k_,Y_k_) are the coordinates of each boundary pixel, *k.* After the centroid value is obtained, the distance of each boundary point to the centroid was calculated using Equation (9). Figure 7 shows the sample of regular and misshapen mango while Figure 8 shows the distance signature R(k) of the regular and misshapen mango.

(9) $$R\left(k\right)=\;\sqrt{{{(x}_{k}-{\;x}_{c})}^{2}{+\;(y}_{k}-{\;y}_{c}{)}^{2}\;}$$

ElMasry [22] linearly normalized the boundary profile to 512 distance points for equal level of comparison. Figure 8b shows the normalized boundary profile for the mango. The R(k) is then subjected to Discrete Fourier Transform (DFT), yielding a one-dimensional feature vector of the Harumanis mango. Then the transformation is mathematically implemented in the Fourier space using Equation (10):

(10) $$\left|F(m)\right|=\;\frac{1}{N}\sqrt{{\left[\overset{N}{\underset{k=0}{\sum }}R\left(k\right)\cos\left(\frac{2\pi \mathrm{mk}}{N}\right)\right]}^{2}\;}+{\left[\overset{N}{\underset{k=0}{\sum }}R\left(k\right)\sin\left(\frac{2\pi \mathrm{mk}}{N}\right)\right]}^{2}$$

The descriptors are influenced by the curve shape and the initial point of the curve. Thus, calculating and examining each harmonic component in |F(m)| provides an indication of the shape. The plot of Fourier descriptors produces a pattern or fingerprint which uniquely describes this shape. In theory, the order of Fourier descriptors ranges from zero to infinity [26]. Hence, only the first few segments of |F(m)| are recognizable and generally required to discriminate the difference between mango shapes. In order to extract the information of the shape effectively, a method of harmonics, F(m) multiplied by its magnitude, m was used to obtain the boundary profile of the mango. F(m) × m can be interpreted as the derivative of the boundary signature while F(m) × m^2^ is the curvature of the boundary [22]. The increase power value of h from 1 to 3 contributed to a significant improvement of the higher frequency component which shows the detailed noise in the profile. Hence, parameter S_1_, S_2_ and S_3_ were calculated from the Fourier descriptor using first 10 harmonics to express the shape of mango as in Equation (10) to Equation (13):

(11) $$S_{1}=\overset{10}{\underset{m=0}{\sum }}F(m)\;\times \;m$$

(12) $$S_{2}=\overset{10}{\underset{m=0}{\sum }}{F(m)\;\times \;m}^{2}$$

(13) $$S_{3}=\overset{10}{\underset{m=0}{\sum }}{F(m)\;\times \;m}^{3}$$

### 2.6. Volume and Mass Evaluation Using Image Processing

**Figure 9:**
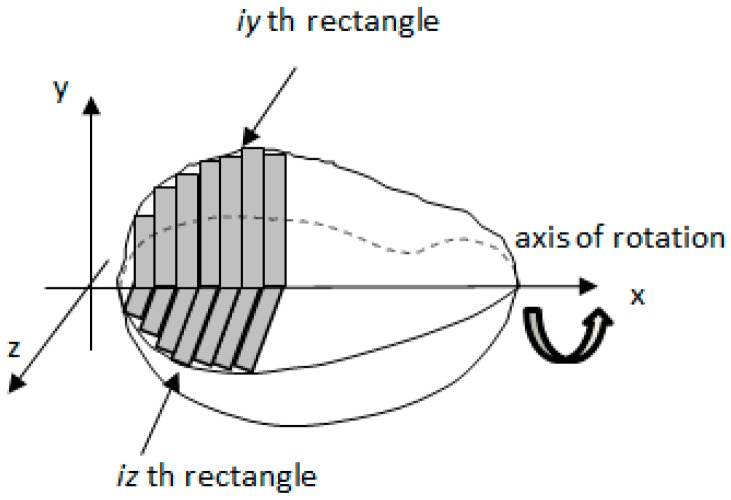
The outline image of Harumanis mango was assumed to be composed of individual rectangular elements.

**Figure 10:**
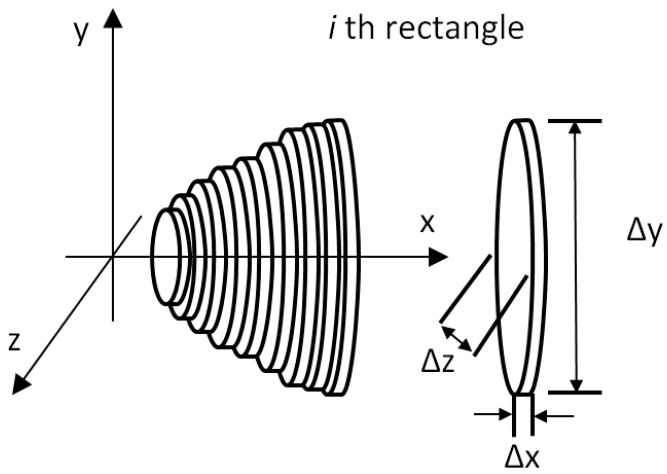
Revolving each rectangular element around x-axis generated an elliptic cylindrical disk.

The boundary of the side view image and top view image was used to calculate the total volume of the mango by using the disk method [27]. Images of the regular mango were calculated for its volume while images of the misshapen mango were discarded. Both mangoes boundary image were combined to obtain a three-dimensional measurement. Each three-dimensional outline image was considered to be the sum of individual rectangular elements as shown in Figure 9. A cylindrical disk with elliptic shape was produced by revolving each rectangular element around the x-axis as shown in Figure 10. 

The volume of each disk calculated by using Equations (14) and (15). Equation (14) shows the volume, V_i_, of one cylindrical disk while Equation (15) shows the cross-sectional area, A_i_, of the same disk:

V_i_ = A_i_∆x_i_ (14)

A_i_ = π(∆y_i_/2)(∆z_i_/2) (15)

A program was developed using the LabVIEW platform to calculate the area, volume, and sum of all disks volumes. An algorithm was used to obtain the measurements of the mango using the boundary image. Each cylindrical disk was considered to have a thickness of 1 pixel. Based on the diameter of the major axis and minor axis, the volume of each disk was calculated. The total sum value of these volumes was used to obtain the estimated volume by using Equation (16):

(16) $$V_{\mathrm{total}}=\;\overset{n}{\underset{i=1}{\sum }}V_{i}$$

The total volume was then used to calculate the mass of each regular mango. The regular mango was categorized into its grade based on the standard set by the Perlis Agriculture Department as in Table 1. Grade A is the highest grade of mango which mass is more than 400 g, while grade B mass is between 351 g and 399 g. Mangoes which mass are lower than 350 g are categorized as grade C. The mangoes were pre-classified into these grades and the results from the grading system were evaluated.

## 3. Results and Discussion

### 3.1. Measurement Calibration Results

**Figure 11:**
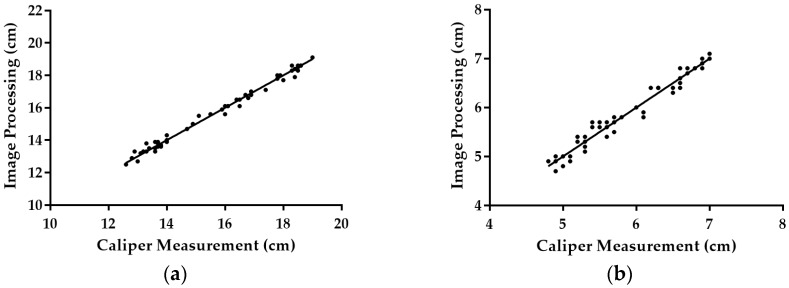
Dimension measurement of mango using caliper and image processing. (**a**) Plot for length measurement; (**b**) Plot for height measurement.

**Figure 12:**
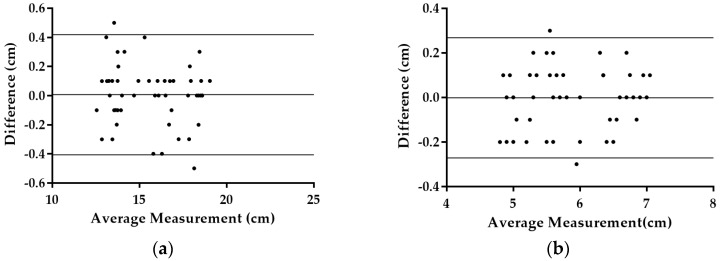
Bland-Altman plot for comparing difference and average of water displacement method and disk method; outer line indicate 95% limits of agreement and center line show the average difference. (**a**) Plot for length measurement; (**b**) Plot for height measurement.

Fifty random mangoes were selected from among the samples for measurement calibration. The number of pixels representing the maximum length and maximum height of the mango were measured on the captured image. These values were used in Equations (2) and (3) to get the estimated maximum length and maximum height of the mango. Plots of the maximum length and maximum height measured by caliper and image processing are shown in Figure 11. The coefficient of determination (R^2^) of both length and height were 1.000 and 0.993, respectively. R^2^ provides a measure of how well observed outcomes are replicated by a model, based on the proportion of total variation of the outcomes. The closer the R^2^ value is to 1 means the image processing measurements are closer to the caliper measurements. The proposed equation of the image processing method yielded above 99% accuracy in estimating mango fruit dimensions. Figure 12 shows the graph for the Bland-Altman method. The plot indicates that the dimension measurement difference between these two methods was normally distributed. The 95% limits of agreement for measurement difference were expected to be scattered between d – 1.96 sd and d + 1.96 sd. The outer line of Figure 12 indicates the 95% limits of agreement while the center line indicates the mean difference. The 95% limits of agreement for comparison of image processing measurement and caliper measurement for length and height were (−0.406:0.418) and (−0.272:0.268), respectively.

### 3.2. Shape Features Analysis

The first stage of analysis is to determine the uniformity of the shape which is categorized into regular shape and misshapen. As for the second stage, mangoes were classified according to their grade based on the mass value. All mango samples were inspected and classified first by human experts based on their shape. Approximately 180 fruits were sampled, of which 21 fruits were classified as misshapen, while the remaining 159 were classified as regular shape. The value of mean and standard deviation of all shape features which extracted from the dataset of image are shown in Table 2. From the result, misshapen mangoes have a wide range of variation compared to the regular mangoes. This assumption was based on the high value of the standard variation for each feature for misshapen mangoes. 

Size-dependent features and Fourier dependent features were used in the algorithm for detecting the shape of the mangoes. Fourier dependent features use the Fourier transform equation to calculate the magnitude of the mango’s polar signature extracted from the boundary pixels. Different values of Fourier’s harmonic component bear different shape information which could be used to explain the irregularity of the mango shape. Thus, it was used to calculate the different harmonic component of the mango and the result indicated that misshapen mangoes have higher values of |F(m)| compare to the regular-shaped mangoes.

### 3.3. Discriminant Analysis

Discriminant analysis is basically used to identify a subset of dominant features which are responsible for splitting a set of observations into two or more groups. The successful application of shape recognition using machine vision depends on the selection of a decent range and number of features employed in the calibration model. Stepwise discriminant analysis was applied in this research. Wilks’ Lambda analysis was chosen to select the best data subset which contains the shape-dependent features and Fourier dependent features. This method will analyze the data subset; and recognize the insignificant and highly insignificant or potent features. Variable selection of Wilks’ Lambda method conduct in an iterative way, systematically removing features variable whose F-statistics which are smaller than F-to-remove and retaining those value whose F-statistics are greater than F-to-enter values [26]. 

Stepwise discriminant analysis was implemented to pinpoint a subset of dominant shape features (Aspect ratio, Area ratio, Roundness, S_1_, S_2_ or S_3_) which most discriminate mango shape groups. Summary of shape variable selection is shown in Table 3. At each step, the variable that minimizes the overall Wilks’ Lambda is entered. It was noticeable from Table 3 that the model only accepts four features (S_1_, S_2_, S_3_ and Area Ratio) and removes the other two features from the final model. 

Table 4 shows the classification of the mangoes using the developed model of discriminant analysis for the training set. The features selected by the stepwise discriminant analysis were able to predict the mango shape with a success rate of 98.3%. The model was able to predict the regular shape of mangoes with 99.4% success rate, while the rate for the misshapen mangoes is 90.5%. For the next step, a model was created based on a classification function in order to reduce the error of predicting the mango shape in each respective group. Two classification functions were created using Fisher’s linear discriminant function to explain each group. These functions were implemented on model and tested on each mango and the result was used to determine which group of the mango belongs to. Equations (17) and (18) were the functions derived from the model based on the values in Table 5. Both equations then were applied to the system to predict the shape of unknown mangoes.

Regular = −198.85 + 24.96 × S_1_ – 8.95 × S_2_ + 0.78 × S_3_ + 0.34 × Area Ratio (17)

Misshapen = −228.38 + 22.05 × S_1_ – 6.97 × S_2_ + 0.59 × S_3_ + 0.37 × Area Ratio (18)

These final linear equations were tested on the real-time system to predict the mango shape regularity. The equations were applied to the system to predict the shape of a set of randomly selected mangoes which consisted of 126 regular mangoes and 14 misshapen mangoes. This step was done to verify the performance of the mango shape classification during inline operations. Only the top view of mango was used to determine the shape class. The mango images were analyzed directly after the image capturing process. The obtained features values from the image were then used by the algorithm to predict the shape of mango. Table 6 shows the performance of the algorithm in classifying the randomly selected mangoes. The result shows that the integration of size-dependent features and Fourier dependent features can predict the shape of mangoes with an acceptable rate of accuracy which was 98.4% for regular and 85.7% for misshapen ones. 

### 3.4. Comparison of the Disk Method with Water Displacement

**Figure 13:**
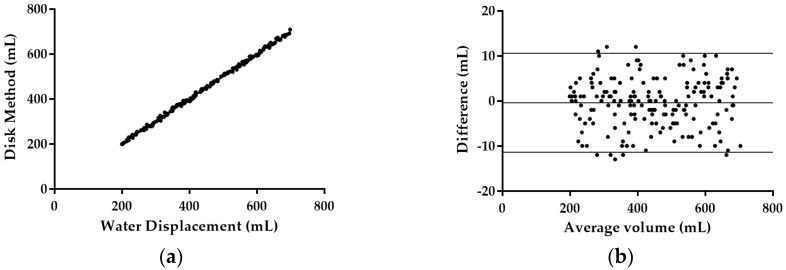
(**a**) Plot of mango volume measure by disk method estimation and water displacement method with regression line (R^2^); (**b**) Bland-Altman plot for comparing difference and average of water displacement method and disk method; outer line indicate 95% limits of agreement and center line show the average difference.

A plot of the volumes measured by image processing using the disk method and water displacement is shown in Figure 13a. The coefficient of determination (R^2^) value was 0.9985. 

Mean volume difference between both methods was d = −0.400 mL where the 95% confidence interval ranged from −1.224 mL to 0.4241 mL. Standard deviation for the volume difference was s = 5.603 mL. The paired samples *t*-test result showed that mango volumes estimated using the disk method were not significantly different from the volumes measured using water displacement (P = 0.3394) as indicated in Table 7. Figure 13b shows the plot for the Bland-Altman method. The plot indicates that the volume differences between these two methods were normally distributed. The 95% limits of agreement for volume difference were expected to lie between d – 1.96 sd and d + 1.96 sd. The outer line of Figure 13b indicates the 95% limits of agreement while center line indicates the mean difference. The 95% limits of agreement for the comparison between volumes measured using the water displacement method and the volumes calculated using disk method were −6.995 mL and 15.490 mL.

From these results, it can be stated that mango size did not have any effect on the accuracy of the estimated volume which the P value was higher than 0.05. The overestimation problem which been state in Koc’s [12] research seems to be solved using the method proposed in this research. The combination of a more accurate dimension measurement method and a two-dimensional disk method could successfully determine the accurate volume of Harumanis mangoes. 

### 3.5. Estimation of the Mass of Harumanis Mango

**Figure 14:**
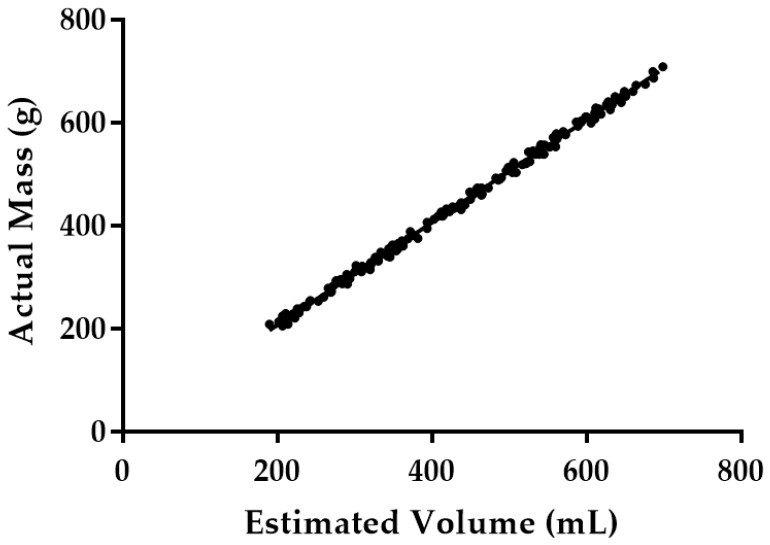
Plot of Harumanis mango’s mass and estimated volume.

In-line measurement of fruit mass using physical sensors is a time consuming method. Another alternative method like volume-based sorting might reduce the processing time and can be more efficient than conventional weight sorting. A fruit’s mass can be estimated from its volume if the density of the fruit is assumed constant. Density can vary during the maturation process but the variation is small and unpredictable [11]. Thus, the usage of weighing devices can be eliminated from the packaging line. Spreer [28] and Schulze [29] determined the mass of the Chok Anan mangoes and Nam Dokmai mangoes using the physical properties of the mango. Relationships between the physical traits were determined and a high correlation between the mass and the physical traits was found. Sa’ad [24] obtained a high correlation value between Harumanis mango volume estimated using the cylinder method and its weight with a value of R^2^ = 0.94. In this research, the estimated volume was plotted against the actual mass as shown in Figure 14. It shows a high correlation between the two values with R^2^ = 0.9978. This high R^2^ value indicates that the linear formula can be used to estimate the mass of the mango based on the estimated volume. Equation (19) was the linear formula generated from the graph in Figure 14.

Mango Mass = 0.9973 × Estimated Volume + 6.778(19)

The estimated mass equation was tested on the on-line system to evaluate the accuracy of the classification based on its weight. The correct classification rate of the Harumanis mango by mass obtained from the proposed method was 94.4%. Table 8 shows the confusion matrix for the validation set (126 regular mangoes). The classification errors may occur because of the estimated mass falls in between the grade categories and errors in determining the exact dimensions of mangoes because of dirt and bruises on the skin. 

## 4. Conclusions

In this work, an image processing techniques to classify Harumanis mangoes based on shape and mass analysis were developed and presented. An image capturing platform was designed and constructed to obtain the images from two different viewpoints. The platform has also been used to test the algorithm’s ability to classify mangoes based on their shape. An algorithm was developed to grab and process the mango images in real time while the mangoes travel along the high-speed conveyor. Stepwise discriminant analysis was used to identify the most effective shape parameters for detecting misshapen mangoes. Three parameters calculated from the Fourier descriptor of the boundary signature of mangoes along with the area ratio size-shape parameters were identified to be effective in describing mango shapes. The in-line assessment of the proposed method showed that the accuracy for detecting mango shapes was 98%. 

Another algorithm was developed to automatically calibrate the mango measurement each time a new image was captured. The method used the known distance from the camera to the object plane and related information which extracted from the image. The information was then used to determine the exact measurement of the mango. The relation between the caliper measurement and image processing measurement was high where the R^2^ values for the length and height measurement were 1.000 and 0.993, respectively.

Next the algorithm was used to determine the mass of the mango. Top and side views of mango were used to estimate the mango volume by using the disk method. The volume estimated using the disk method was compared with the volume measured using the water displacement method. The difference between the volumes estimated using this technique was not statistically significant (P > 0.05). The estimated volume was then used to estimate the mass of the mango. The R^2^ value between the estimated volume and actual mass was high (R^2^ = 0.94), thus indicating that there was a strong relationship between those two values. A prediction model was fitted with this algorithm to estimate the mass and tested on the in-line system. The classification result showed an accuracy of 94% when grading mangoes. 

From the results, it can be concluded that the proposed integration between Fourier-shape parameters and size-shape parameters was very effective in classifying the mango shape. The combination between an image calibration method, volume estimation method, and mass estimation method has been helpful to classify mangoes according to their grade. This system can be applied at an industrial scale but some modifications need to be done. The image processing method can be improved in order to detect other features such as bruises, defects, cuts and disease. The process of image acquisition could be sped up by adding a frame grabber to the system.

## Figures and Tables

**Table 1:** Market grading standards for Harumanis mango set by the Perlis Department of Agriculture.

Grade	Qualitative Measure
A	Standardized in shape and size (mass > 400 g)
B	Standardized in shape and size (mass 351–399 g)
C	Standardized in shape and size (mass < 350 g)
Rejected	Not standardized in shape and size

**Table 2:** Shape features (means + standard deviation) of regular and misshapen mango.

Feature	Shape Parameter	Regular	Misshapen
**Size-shape parameters**	Aspect Ratio	1.66 ± 0.11	1.58 ± 0.11
	Area Ratio	593.79 ± 56.79	653.66 ± 69.18
	Roundness	0.73 ± 0.03	0.67 ± 0.05
**Fourier-shape**	S_1_	20.49 ± 2.03	25.72 ± 5.094
	S_2_	62.47 ± 9.75	94.25 ± 26.21
	S_3_	311.29 ± 68.01	510.31 ± 190.27

**Table 3:** Summary of shape features by discriminant analysis.

Step	Entered	Removed	F
**1**	S_2_		0.605
**2**	Roundness		0.498
**3**	Area Ratio		0.459
**4**	S_3_		0.442
**5**	S_4_		0.425
**6**		Roundness	0.426

**Table 4:** Confusion matrix of discriminant model for predicting the shape of training set mangoes.

From/To	Misshapen	Regular	Total	Percentage
Misshapen	19	2	21	90.5%
Regular	1	158	159	99.4%
Total	20	160	180	98.3%

**Table 5:** Classification functions coefficient derives from the discriminant model for predicting mango shape.

Shape Features	Misshapen	Regular
S_1_	22.05	24.96
S_2_	−6.97	−8.95
S_3_	0.59	0.78
Area_Ratio	0.37	0.34
(Constant)	−228.38	−198.85

**Table 6:** Classification accuracy of the shape detection algorithm in on-line operation for sorting shape in testing set.

Actual/Predicted	Regular	Misshapen
Regular	98.4%	1.6%
Misshapen	14.3%	85.7%

**Table 7:** Comparison of the two volume measurement methods by paired *t*-test.

Paired *t-*test	95% Confidence Level of the Mean Difference
P = 0.3394	−1.224 and 0.4241

**Table 8:** Confusion matrix of mango grading in the proposed on-line system. The misshapen mangoes were removed from the size grading process. The regular mangoes were graded cording to pre-defined thresholds.

Actual/Predicted	Grade A	Grade B	Grade C	% Correct
Grade A	40	2	0	95.2
Grade B	1	39	2	92.9
Grade C	0	2	40	95.2
Total	41	43	42	94.4
